# Abeta(1-42) Enhances Neuronal Excitability in the CA1 via NR2B Subunit-Containing NMDA Receptors

**DOI:** 10.1155/2014/584314

**Published:** 2014-09-03

**Authors:** Edina Varga, Gábor Juhász, Zsolt Bozsó, Botond Penke, Lívia Fülöp, Viktor Szegedi

**Affiliations:** ^1^Department of Medical Chemistry, University of Szeged, Szeged 6726, Hungary; ^2^Biological Research Center—Biochemistry, Hungarian Academy of Sciences, Temesvari Körút 32, Szeged 6726, Hungary

## Abstract

Neuronal hyperexcitability is a phenomenon associated with early Alzheimer's disease. The underlying mechanism is considered to involve excessive activation of glutamate receptors; however, the exact molecular pathway remains to be determined. Extracellular recording from the CA1 of hippocampal slices is a long-standing standard for a range of studies both in basic research and in neuropharmacology. Evoked field potentials (fEPSPs) are regarded as the input, while spiking rate is regarded as the output of the neuronal network; however, the relationship between these two phenomena is not fully clear. We investigated the relationship between spontaneous spiking and evoked fEPSPs using mouse hippocampal slices. Blocking AMPA receptors (AMPARs) with CNQX abolished fEPSPs, but left firing rate unchanged. NMDA receptor (NMDAR) blockade with MK801 decreased neuronal spiking dose dependently without altering fEPSPs. Activating NMDARs by small concentration of NMDA induced a trend of increased firing. These results suggest that fEPSPs are mediated by synaptic activation of AMPARs, while spontaneous firing is regulated by the activation of extrasynaptic NMDARs. Synaptotoxic Abeta(1-42) increased firing activity without modifying evoked fEPSPs. This hyperexcitation was prevented by ifenprodil, an antagonist of the NR2B NMDARs. Overall, these results suggest that Abeta(1-42) induced neuronal overactivity is not dependent on AMPARs but requires NR2B.

## 1. Introduction

Extracellular recording from hippocampal slices has long been a method of choice for determining the changes in excitability and synaptic plasticity of the CA1 microcircuitry [1]. Spontaneous spikes and, far more often, field excitatory postsynaptic potentials (fEPSPs) are recorded extracellularly from a local network of neurons; however, the relationship between these two phenomena is not fully clear. Intuitively, both electrophysiological events correlate with the excitability of the network under investigation, but these events are generated by different mechanisms. Field EPSPs are mainly composed of subthreshold events from a population of neurons, like dendritic depolarizations [2] and glial contributions to the net extracellular charge-flow, arising mainly from the function of transporters [3, 4]. In contrast, spontaneous firing represents only neuronal suprathreshold events [5]. Another difference is that a fEPSP is evoked by a stimulus, and therefore it is the result of a coordinated and synchronized electrical activity of a cell population, mediated by synaptic connections. Spontaneous spikes, in contrast, are not evoked by an external stimulus, and they are more likely to be dependent on intrinsic network connections and properties [6].

N-Methyl-D-aspartate receptor (NMDAR) and *α*-Amino-3-hydroxy-5-methyl-4-isoxazolepropionic acid receptor (AMPAR) play a key role in generating rapid excitatory events in the CA1, but these receptors serve different purposes. Their relative contribution to fEPSPs is well described [7]; however, their involvement in spontaneous spike-generation remains unknown and data on the correlation of extracellularly recorded field responses and action potentials are scarce. Activation of glutamate (Glu) receptors is known to play an important role in the mechanisms of neuronal plasticity that might be the cellular basis of learning and memory [8]. Several studies have shown that the synaptic plasticity is impaired in Alzheimer's disease (AD) [9], the most common form of neurodegenerative disease, characterized by the presence of insoluble amyloid deposits in the brain. Overwhelming evidence suggests that the main component of plaques, amyloid-beta (Abeta) peptide, is thought to be responsible for the synaptic and cellular pathology of AD (for review see [10]). Numerous studies have reported that the severity of AD strongly correlates with decreased synapse density in hippocampus and cortex and with disruption of memory-related synapse function. Literature data strongly support the synaptotoxicity of Abeta(1-42), and the accumulation of soluble Abeta(1-42) [11] in the brain of patients and animal models of AD is associated with impairments of cognition and memory [12–14]. Recently, Abeta(1-42) was shown to induce epileptiform activity, both* in vitro* [15] and* in vivo*, and transgenic mice overexpressing Abeta develop seizures over time [16]. Moreover, AD patients have an estimated 87 times higher probability of developing epileptic seizures compared to age-matched population [17]. Despite the recent advancement in the research of the pathomechanisms of AD, the exact mechanism by which neuronal overactivity develops is unknown.

Recent findings suggest that Abeta blocks neuronal glutamate (Glu) uptake at synapses, leading to increased Glu level at the synaptic cleft [18]. Increased brain extracellular Glu, but not *γ*-aminobutyric acid (GABA), concentration was found upon Abeta administration by use of microdialysis technique [19, 20]. A resultant rise in Glu levels would lead to a spillover and activation of extra- or perisynaptic NMDARs enriched in the B isoform of the NMDAR 2 subunit (NR2B), which play a major role in the induction of long-term depression (LTD) [21] and have also been shown to help mediate the inhibition of long-term potentiation (LTP) by soluble Abeta oligomers [22].

In this study we investigated the mechanisms by which fEPSPs and spontaneous firing are regulated in the CA1 of hippocampal slices. We confirmed that fEPSP, but not spiking activity, is mainly mediated by AMPARs. In contrast, spontaneous activity is regulated by NMDARs. Bath application of synaptotoxic Abeta(1-42) greatly enhanced spontaneous firing rate, which could be prevented by blocking NR2B subunits.

## 2. Experimental Procedures

### 2.1. Compounds

For the preparation of artificial cerebrospinal fluid (ACSF), all salts, glucose, 6-cyano-7-nitroquinoxaline-2,3-dione (CNQX), (+)-MK-801 hydrogen maleate, and *α*-(4-Hydroxyphenyl)-*β*-methyl-4-benzyl-1-piperidineethanol (+)-tartrate salt (ifenprodil) were purchased from Sigma-Aldrich (St. Louis, MO).

### 2.2. Animals

The study conformed to EU Directive 2010/63/EU and was approved by the regional Station for Animal Health and Food Control under Project License XVI/8/2013. BALB/c mice were housed in groups of 2-3 under standard conditions (24°C, 12 h light-dark cycle) with food and water available* ad libitum*.

### 2.3. *Ex Vivo* Electrophysiology

Hippocampal slices of 400 *μ*m in thickness were prepared from the brains of 3-month-old mice using a standard protocol [23]. Briefly, slices were incubated in artificial cerebrospinal fluid ACSF gassed with 95% O_2_ and 5% CO_2_ at 35°C for 60 min. ACSF was composed of (in mM) 130 NaCl, 3.5 KCl, 3 CaCl_2_, 1.5 MgSO_4_, 0.96 NaH_2_PO_4_, 24 NaHCO_3_, and 10 D-glucose, pH 7.4. Individual slices were transferred to a 3D-MEA chip with 60 tip-shaped and 60 *μ*m high electrodes spaced by 200 *μ*m (Qwane Biosciences, Lausanne, Switzerland). The surrounding solution was quickly removed, and the slice was immobilized by placing a grid onto it. The slice was continuously perfused with oxygenated ACSF (3 mL/min at 36°C) throughout the entire recording session. Unfiltered data were recorded using a standard, commercially available MEA 60 setup (Multi Channel Systems MCS GmbH, Reutlingen, Germany). Field EPSPs were recorded from the proximal stratum radiatum at 5 kHz, while spontaneous spiking activity was recorded from the CA1 stratum pyramidale at a frequency of 25 kHz for 5 min epochs. For analyzing and sorting the spiking activity, Spike2 software package (Cambridge Electronic Design, Cambridge, UK) was used. Recordings were filtered between 300 and 3000 Hz offline, and the threshold for spike detection was 2.5 fold higher than the noise level. The data of those electrodes were included in the analysis where the initial spiking activity was above 0.5 Hz. Firing activity ranged from 150 to 1000 spikes/5 min. Data are considered as multiunit activity.

#### 2.3.1. Stimulation Protocol

The Schaffer-collateral was stimulated by injecting a biphasic voltage waveform (−100/+100 *μ*s) through one selected electrode at 0.033 Hz. Care was taken to place the stimulating electrode in the same region at every slice. The peak-to-peak amplitudes of fEPSPs at the proximal stratum radiatum of CA1 were analyzed. After a 30 min incubation period, the threshold and maximum stimulation intensities for evoked responses were determined. To evoke responses, 30% of the maximal stimulation intensity was used. The level of LTP (the last 10 peak-to-peak amplitudes) was compared to the average of the last 20 peak-to-peak amplitudes of evoked fEPSPs before applying theta-burst stimulation (TBS). TBS comprised of 15 bursts given at 5 Hz and individual burst contained 4 pulses given at 100 Hz per burst.

#### 2.3.2. Drug Treatments

**Figure 1:**
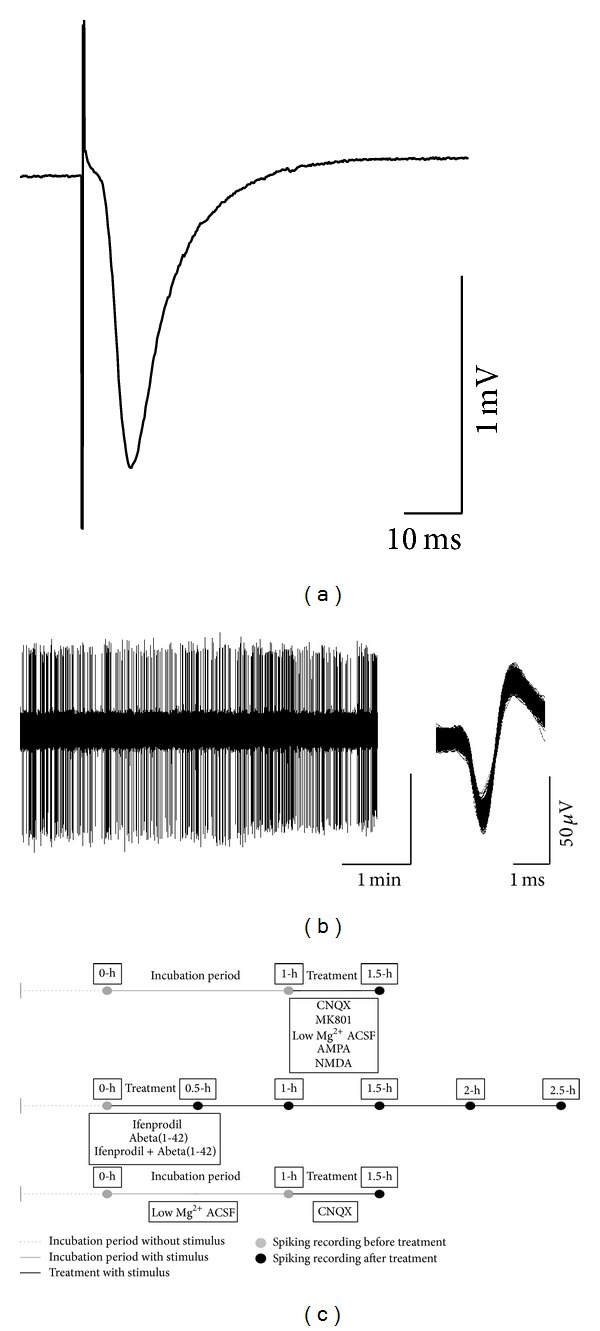
Exemplar recordings and the timeline of the experiments. Both evoked fEPSPs and spiking activity recording electrodes were located in the CA1 region of mouse hippocampal slice. The fEPSPs were recorded from the stratum radiatum (a) and the spontaneous spiking was recorded from the stratum pyramidale (b). Both fEPSPs and spiking activity were recorded from the same slice. Data were considered as multiunit activity. (c) shows the timeline of the recordings. The initial spiking activity (recorded at 0 h) was considered as 100% in each slice.

All slices were incubated for 30 min without stimulation in the recording chamber before any recording was done. Following the first spike-recording, slices were left to incubate a further 60 min with continuous fEPSP recording and then slices were treated with 10 *μ*M CNQX or 10 *μ*M MK801 or 25 *μ*M MK801 or ACSF containing low concentration of Mg_2_SO_4_ (low Mg^2+^ ACSF; 0.25 mM) or 0.5 *μ*M NMDA or 0.05 *μ*M AMPA for 30 min (see Figure 1(c)). Spontaneous activity was recorded before and 30 min after treatment for 5 min and the spiking data was compared to the untreated group recorded at the same time point. Other cohorts of slices were treated with 1 *μ*M oligomer Abeta(1-42), ifenprodil (3 *μ*M), and ifenprodil + Abeta(1-42) for 2.5 h. The spiking activity was recorded at every 30 min for 5 min. The effect of CNQX was also tested in low Mg_2_SO_4_-containing ACSF (0.25 mM), where modified ACSF was perfused to the slices from the beginning of the experiment. Electric stimulation was stopped during spontaneous activity recordings. Spiking frequency of each slice (number of spikes) was normalized to the initial firing activity (following the first 30 min incubation; 0 h), which was taken as 100% in all channels. Following this, slices were treated with 1 *μ*M oligomer Abeta(1-42) for 60 min and then LTP was induced by the TBS protocol. LTP was followed for 90 min after TBS. During treatment, evoked fEPSPs were recorded.

### 2.4. Synthesis and Characterization of Abeta(1-42)

Detailed description of the synthesis and characterization of Abeta(1-42) is reported in [23, 24]. Briefly, a depsipeptide derivative of Abeta(1-42) was synthesized and, after purification, it was used in lyophilized form. A 200 *μ*M stock solution of the peptide was prepared in 0.1 mM NaOH, and the pH was set to 11.0. After incubation for 2 h at ambient temperature, the stock solution was diluted into ACSF to a concentration of 50 *μ*M and the pH was set to 7.3. The peptide solution was incubated for 12 h at 37°C and, prior to use, it was diluted with ACSF to a final concentration of 1 *μ*M. Aggregation grade and the size distribution of the oligomers were checked by Western blots by following the methods described in [23]. Oligomers were detected either with sequence-specific BAM10 antibody (Sigma-Aldrich) or with conformation specific OC antibody (Millipore), which detects the oligomers of fibrillar nature, that is, with beta sheet structure.

### 2.5. Statistics

#### 2.5.1. Statistical Analysis for fEPSPs

Testing for normality was done with the Kolmogorov-Smirnov normality test. Our data have shown normal distribution; hence, independent-samples *t*-test and one-way repeated measures analysis of variance (ANOVA) were used with the Bonferroni test for* post hoc* analysis. The *P* value ≤ 0.05 was considered significant in all cases. Data were analyzed using SPSS statistical software.

#### 2.5.2. Statistical Analysis for Spiking Rate

Kolmogorov-Smirnov test was used also for testing normality of these data. Since our data have shown nonparametric distribution, we used nonparametric tests (Kruskal-Wallis test was followed by Mann-Whitney *U* test) for determining differences between two and several groups. Data were analyzed using SPSS. *P* value of ≤ 0.05 was considered significant in all cases.

## 3. Results

### 3.1. Field EPSPs, but Not Spontaneous Spikes, Are Mediated by AMPARs

We recorded fEPSPs from the stratum radiatum (Figure 1(a)) and in parallel spontaneous spikes from the stratum pyramidale (Figure 1(b)) of the CA1. Both fEPSPs and spontaneous activity could be abolished by application of 1 *μ*M tetrodotoxin (data not shown).

**Figure 2:**
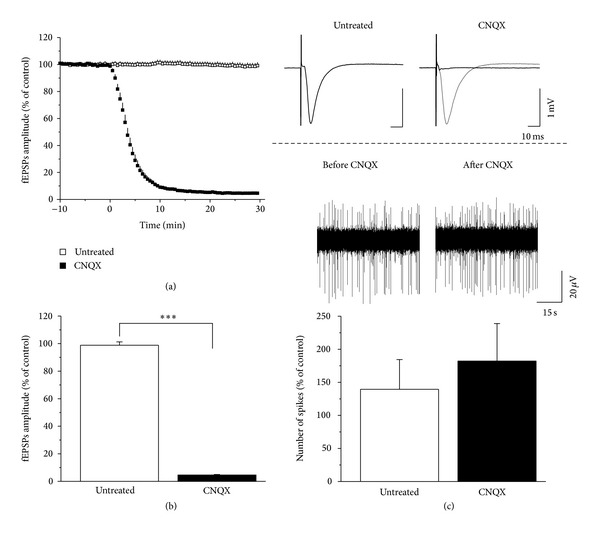
AMPA receptors mediate fEPSPs but not spontaneous activity. Blocking AMPARs abolished fEPSPs (a). Bar graphs show the average of the fEPSPs amplitudes of the 25–30 min period after treatment. CNQX induced a complete reduction of evoked fEPSPs (*n* = 5, *P* < 0.001) (b); however the spiking activity was not affected (c). Inset at the right panel shows representative fEPSPs before (grey) and after (black) treatment and representative spike trains. Error bars show SEM; ***P ≤ 0.001.

First, we investigated the contribution of AMPARs to evoked fEPSPs and spontaneous activity, using CNQX, an AMPAR inhibitor. Blocking AMPARs resulted in a complete reduction of evoked fEPSPs (untreated: *n* = 5; 98.84 ± 2.41% versus CNQX: *n* = 5; 4.62 ± 0.45%, *P* = 0.001, independent samples *t*-test; Figures 2(a) and 2(b)). Conversely, spiking activity remained unchanged (untreated: *n* = 5; 139.55 ± 44.95% versus CNQX: *n* = 5; 182.18 ± 56.76%, Mann-Whitney *U* test; Figure 2(c)), suggesting that AMPARs play a key role in generating fEPSPs but not spontaneous spiking. Under physiological conditions, NMDARs activation is dependent on depolarization, for example, on previous AMPARs activation. Thus we changed to low Mg^2+^ ACSF (0.25 mM) to remove the depolarization-dependent Mg^2+^ plug from the NMDAR. Applying 10 *μ*M CNQX to low Mg^2+^ ACSF slices for 30 min, fEPSPs were completely blocked (control: *n* = 6; 95.54 ± 81% versus CNQX in low Mg^2+^-containing ACSF: *n* = 6; 6.44 ± 0.36%, *P* < 0.001, independent samples *t*-test; see Supplementary Figure 1 available online at http://dx.doi.org/10.1155/2014/584314). We have also tried to activate AMPARs by applying a low concentration of AMPA (0.05 *μ*M), but we observed epileptiform field responses and a huge increase of basal activity which hindered the unambiguous detection of action potentials (Supplementary Figure 2).

### 3.2. Spontaneous Firing, but Not fEPSP, Is Governed by NMDAR Function

**Figure 3:**
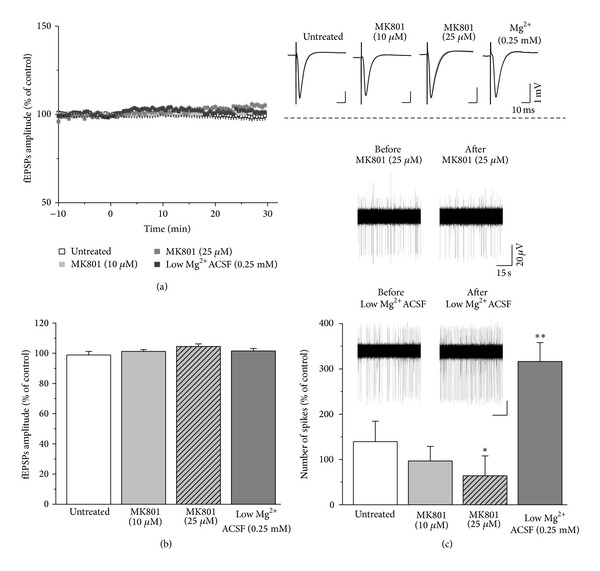
NMDA receptors mediate spontaneous activity but not fEPSPs. Blocking (MK801) or enhancing (low-Mg^2+^ ACSF) NMDAR function did not influence fEPSPs (a). Right panel shows representative fEPSPs before (grey) and after (black) treatment. Bar graphs show the average of the fEPSPs amplitudes of the 25–30 min period after treatment (b). In contrast, MK801 dose dependently reduced spiking frequency and low Mg^2+^ ACSF enhanced firing rate (untreated versus MK801 in 25 *μ*M: *P* = 0.035 and untreated versus low Mg^2+^ ACSF: *P* = 0.004) (c). Inset shows representative spike trains. Error bars show SEM; *P ≤ 0.05, **P ≤ 0.01.

Next, we focused on NMDARs. Applying an NMDAR antagonist, MK801 resulted in a dose-dependent decrease of spiking rate (untreated: *n* = 5; 139.55 ± 44.95% versus MK801 in 10 *μ*M: *n* = 5; 96.73%±32.22, *P* = 0.48; MK801 in 25 *μ*M: *n* = 5; 29.23 ± 22.93, *P* = 0.031, Mann-Whitney *U* test; Figure 3(c)). MK801 did not have any effect on fEPSP in either 10 *μ*M or 25 *μ*M (untreated: *n* = 5; 98.8 ± 2.4% versus MK801 in 10 *μ*M: *n* = 5; 101.23 ± 1.17% versus MK801 in 25 *μ*M: *n* = 5, 104.49 ± 1.72%; one-way ANOVA and Bonferroni* post hoc *test; Figures 3(a) and 3(b)). Another cohort of slices was treated with ACSF having reduced Mg^2+^ concentration for 30 min. We have observed a massively elevated frequency of the spontaneous spiking activity compared to the untreated slices (*n* = 7, 316.34 ± 41.89%, *P* = 0.004, Mann-Whitney *U* test; Figure 3(c)), but evoked fEPSP responses remained unaltered (*n* = 7, 101.54 ± 1.69%, one-way ANOVA and Bonferroni* post hoc* test). Based on these results we hypothesized that spontaneous firing is mediated by the ambient Glu concentration acting on the extrasynaptic NMDARs. Therefore we tried to activate selectively this set of receptors by applying low concentration of NMDA (0.5 *μ*M) in normal ACSF [25]. This treatment resulted in unchanged fEPSPs (untreated: *n* = 5; 98.8 ± 2.4% versus NMDA: *n* = 9; 94.03 ± 1.04%; independent-samples *t*-test) but induced a trend of elevated firing rate (untreated: *n* = 5; 139.55 ± 44.95% versus NMDA: *n* = 9; 253.02 ± 105.9%, *P* = 0.51, Mann-Whitney *U* test; Supplementary Figure 3).

### 3.3. Abeta(1-42) Impairs LTP

**Figure 4:**
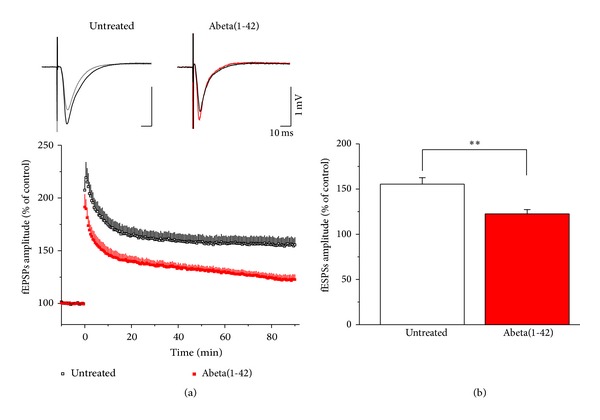
Abeta(1-42) impairs LTP. Representative fEPSPs from both control and Abeta(1-42) groups. Field EPSP was recorded before (grey) and 90 min after LTP induction (control is in black and Abeta(1-42) is in red) from the proximal stratum radiatum of CA1 (see above). Following 1 h of Abeta(1-42) treatment, LTP was induced by TBS protocol. LTP was reduced in Abeta(1-42) treated slices compared to controls 90 min after TBS (untreated versus Abeta: *P* = 0.002) (a). The amplitudes of fEPSPs after TBS were normalized to pre-TBS control. Bar graphs show the average of the last 5 min of LTP. Error bars represent SEM; **P ≤ 0.01 (b).

A major drawback of Abeta studies is that the activity of various Abeta preparations may vary between protocols and even between batches. We verified the activity of Abeta batches we have used for this study by determining their effect on CA1 LTP. Abeta(1-42) was applied for 60 min before inducing LTP by using TBS. Untreated slices showed a robust and permanent elevation of evoked fEPSPs after TBS (*n* = 7; 155.48 ± 7.16% 90 min after TBS), while slices having received Abeta(1-42) failed to exhibit permanent LTP (*n* = 8; 122.54 ± 4.75% 90 min after TBS, *P* = 0.002, independent-samples *t*-test; Figures 4(a) and 4(b)).

### 3.4. Abeta(1-42) Induces Hyperexcitation via NR2B

**Figure 5:**
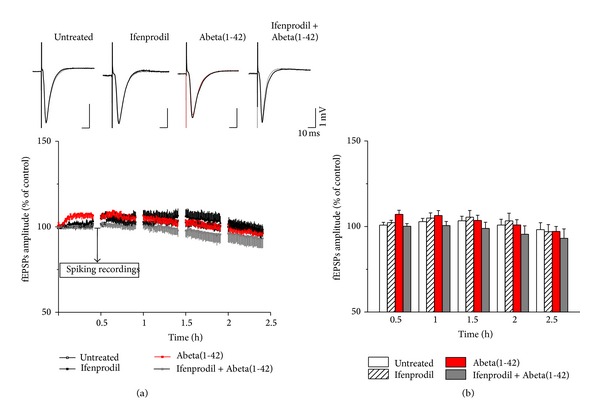
Neither Abeta(1-42) nor ifenprodil affects fEPSPs. Representative fEPSPs are shown before (grey) and after treatment in the upper panel. Neither Abeta(1-42) nor ifenprodil changed fEPSPs amplitudes (one-way repeated measures ANOVA with Bonferroni correction) (a). Bar graphs show the mean of the last 5 min of every recording epoch. Error bars represent SEM (b).

**Figure 6:**
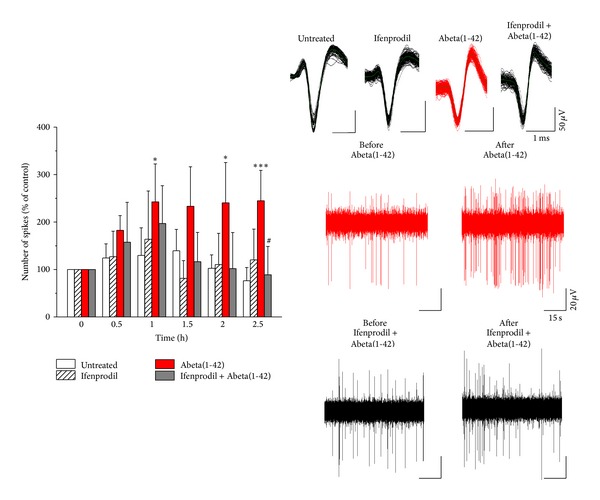
Abeta(1-42) induces hyperexcitation via NR2B. Abeta(1-42) induced increased spiking activity (untreated versus Abeta(1-42): 1 h: *P = 0.043; 2 h: *P = 0.036; 2.5 h: ***P = 0.001; Mann-Whitney *U* test). Ifenprodil did not affect spontaneous activity but prevented the elevated spiking rate caused by Abeta(1-42) (Abeta(1-42) versus ifenprodil + Abeta(1-42) 2.5 h, ^#^ *P* = 0.038; Mann-Whitney *U* test). Upper panel shows exemplar units from the respective recordings, while bottom panel illustrates representative spike trains. Error bars represent SEM; *P ≤ 0.05; ***P ≤ 0.001; ^#^ *P* ≤ 0.05.

Firing rate was determined every 30 min in 5 min epochs within the time frame of the recordings. In the untreated slices the amplitude of fEPSPs increased slightly until 1.5 h reaching 103.36 ± 2.67% of the initial amplitude and then decreased to initial value (*n* = 5; 0.5 h: 100.8 ± 1.65%; 1 h: 102.9 ± 1.84%; 2 h: 100.86 ± 3.38%; and 2.5 h: 98.18 ± 4.04%, one-way repeated measures ANOVA and Bonferroni* post hoc* test; Figures 5(a) and 5(b)). Similarly, spiking frequency did not change over time (*n* = 5; 0.5 h: 124.34 ± 29.72%; 1 h: 129.57 ± 53.28%; 1.5 h: 139.55 ± 44.95%; 2 h: 102.48 ± 28.42; and 2.5 h: 76.16 ± 28.08%, Mann-Whitney *U* test; Figure 6).

Abeta(1-42) applied in 1 *μ*M did not change fEPSPs amplitudes (Abeta(1-42): *n* = 7; 0.5 h: 107.08 ± 2.43%; 1 h: 106.41 ± 2.93%; 1.5 h: 103.54 ± 3.07%; 2 h: 100.92 ± 3.06%; and 2.5 h: 97.1 ± 2.97% compared to untreated slices, see above; Figure 5), suggesting that Abeta(1-42) did not affect the AMPAR-mediated synaptic transmission. On the other hand, Abeta(1-42) induced a massively elevated firing (*n* = 7; 0.5 h: 182.6 ± 30.91%; 1 h: 242.29 ± 83.31%, *P* = 0.043; 1.5 h: 233.29 ± 83.31%; 2 h: 240.32 ± 85.0%, *P* = 0.036; and 2.5 h: 244.72 ± 64.21%, *P* = 0.001, Mann-Whitney *U* test; Figure 6) compared to untreated slices, suggesting that NMDARs are involved in the effect of Abeta(1-42). Several recent reports suggested that the deleterious effect of Abeta is mediated via the NR2B subunit-containing NMDARs (see Section 4). To test whether the observed hyperexcitation in our experimental setup is sensitive to NR2B antagonism, we have applied ifenprodil (3 *μ*M), an antagonist of the NR2B. Ifenprodil did not alter either fEPSPs (ifenprodil: *n* = 5; 0.5 h: 102.26 ± 1.35%; 1 h: 104.95 ± 3.00%; 1.5 h: 105.45 ± 3.91%; 2 h: 103.28 ± 4.53%; and 2.5 h: 96.99 ± 4.15% compared to control, see above; Figure 5) or spiking activity (*n* = 5; ifenprodil: 0.5 h: 127.11 ± 53.62%, 1 h: 163.58 ± 101.77%; 1.5 h: 81.37 ± 37.37%, 2 h: 110.35 ± 65.81%; and 2.5 h: 120.26 ± 64.58%; Figure 6), suggesting that NR2B-activation is not required for basic synaptic transmission in the CA1. However, Abeta(1-42) induced elevated spiking activity was prevented by ifenprodil (ifenprodil + Abeta(1-42): *n* = 5; 0.5 h: 157.44 ± 84.19%; 1 h: 196.87 ± 79.61%; 1.5 h: 116.36 ± 61.71%; 2 h: 101.72 ± 76.26%; and 2.5 h: 88.88 ± 59.67%, *P* = 0.048, Mann-Whitney *U* test compared to Abeta(1-42), see above; Figure 6) without changing of fEPSPs amplitudes (ifenprodil + Abeta(1-42): *n* = 5; 0.5 h: 100.17 ± 1.53%; 1 h: 100.57 ± 2.38%; 1.5 h: 98.85 ± 3.66%; 2 h: 95.45 ± 4.97%; and 2.5 h: 93.15 ± 5.47%; Figure 5), suggesting that the hyperexcitability caused by Abeta(1-42) requires the activation of extrasynaptic NR2B receptors, but not AMPARs.

### 3.5. Characterization of Abeta(1-42)

The size distribution of Abeta(1-42) oligomers formed in 50 *μ*M after incubation at 37°C was studied on Western blots by using two different antibodies; the monoclonal BAM10 antibody is sequence specific and binds to the N-terminal end of the peptide, while OC stains the oligomers of fibrillar nature (protofibrils, oligomers with beta sheet structure) [26]. Supplementary Figure 4 shows the results of the WB experiments. BAM10 staining reveals the presence of both low- and high-molecular-weight oligomers in the sample, while their positive staining with the OC antibody indicates that they have protofibrillar characteristics. The presence of SDS stable dimers and trimers in the sample closely resembles Abeta(1-42) derived from biological sources, namely, from transfected 7PA2 cells [27] and from human brain [28, 29]. These species were shown to have strong synaptotoxic properties [30]. On the other hand, protofibrillar species, which are also abundant in our sample, were also reported to be synaptotoxic [16].

## 4. Discussion

### 4.1. AMPA Receptors Regulate fEPSPs and NMDA Receptors Mediate Spontaneous Spiking

Extracellular fEPSP recordings are the gold standard for determining the excitation and synaptic plasticity in hippocampal slices. The changes of spontaneous spiking activity under conditions that modify network excitability are, however, less well studied in acute slices. In these sets of experiments we have studied the involvement of AMPARs and NMDARs in fEPSPs evoked by Schaffer-stimulation and in spontaneous firing in the CA1. We show that these two electrophysiological markers, previously thought to underlie measures of excitation, are not correlated. Importantly, the spikes we recorded here were almost exclusively localized to the pyramidal layer of CA1; hence, they were presumably from principal cells. Multiunit activity recordings do not allow separating the firing from individual neurons and may include bursts of spikes fired by the same neuron. Thus, in our study, spiking represents the global amount of activity within this network and most probably the activity of principal cells. The firing rates observed in this study were comparable to previously reported values in slices of CA1 of the guinea pig (0.22 and 1.8 spikes/sec) [6]. Under our conditions, blocking AMPAR with CNQX inhibited fEPSPs, however, the spiking activity did not change. In contrast, modulating NMDAR function affected spontaneous firing without any change in evoked fEPSPs. Blocking NMDARs ablated, while enhancing NMDAR function increased spontaneous activity.

### 4.2. Possible Involvement of the CA1 Local Microcircuitry in the Regulation of Spontaneous Spiking

Direct comparison of data from CA3 spiking regulation and our CA1 data is difficult, because glutamatergic pyramidal cells of the CA3 form massive recurrent loops, a feature that is missing in the CA1. Therefore, the activity of CA1 pyramidal cells is regulated by the excitation arriving from CA3 through the Schaffer-collateral and by the inhibition of the local interneuron microcircuitry. Although the activity of CA1 inhibitory cells has been reported to be scarce, compared to CA3 in acute slices, powerful pyramidal somatic inhibition could be detected upon minimal electric stimulation [31], showing that the inhibition is very effective. What is the driving force of the interneuronal activity? One possible explanation is that the spontaneous activity of the incoming axons of the CA3 principal cells recruits a CA1 inhibitory microcircuitry via the Schaffer-collateral. This is unlikely, because there is no correlation between CA3 multiunit activity and the extracellularly recorded inhibitory postsynaptic potential in the CA1 [31]. Another possibility is that the spontaneous discharge of CA1 interneurons, although scarce, is keeping a strong blockade of the principal cells. Favoring this hypothesis, the resting membrane potential is more hyperpolarized in CA1 compared to CA3 pyramidal neurons [32, 33]. These spontaneous discharges might be regulated by the ambient Glu concentration via NMDARs. Indeed, tonic activation of NMDARs by ambient glutamate has been described in virtually all pyramidal cells of the CA1 of the hippocampus in slice models [34–37]. Importantly, as Le Meur et al. have shown [38], neither AMPA/kainate receptors nor metabotropic glutamate receptors contribute to this tonic excitation of pyramidal neurons. This tonic current is not dependent on vesicular release of transmitters from neurons but is affected by inhibition of the enzyme converting Glu, in glutamine in glial cells, indicating that ambient Glu is mainly of glial origin. Consistent with these findings, we have also shown that spontaneous spiking is driven by NMDAR activity, but not by AMPARs, indicating that the tonic activation could reach the threshold of spike generation. The question arises as follows: why do not AMPARs mediate spontaneous activity? NMDARs have a much higher affinity for Glu than AMPARs do; in steady-state conditions, the EC50 for Glu at NMDARs is over two orders of magnitude lower than that at AMPARs [39]. Extrasynaptic NMDARs containing the NR2B subunit have even higher affinity than synaptic NR2A-containing receptors [40]. It should be noted that evoked firing, which is usually detected in the form of population spikes (pop-spikes for short) in CA1 extracellular recordings, is mediated by AMPAR signaling [41]. Under our conditions, action potentials were not inhibited by AMPAR blockade. The reason behind this discrepancy might be that evoked pop-spikes require a synchronous discharge of a population of cells. This coordinated function might be based on the fast AMPAR function.

### 4.3. Abeta(1-42) Impairs LTP and Hyperexcites without Altering fEPSPs in the CA1 

Neuronal hyperexcitability in early AD and in AD modeling mice is an emerging finding that points to a network dysfunction as a critical component of the pathomechanism. Soluble Abeta species have been increasingly implicated as the key pathologic components of the disease, thus elucidating the mechanisms by which these species alter neuronal function is important. We confirmed that the Abeta(1-42) preparation we use is really synaptotoxic, impairing LTP. Next we tested its effect on spontaneous firing and found that it enhanced the rate of spontaneous discharges but did not alter evoked fEPSPs. These results suggest that NMDARs are involved in the hyperexcitability induced by Abeta(1-42). Similar results have been recently reported by Varghese et al. using cultured embryonic rat hippocampal cells. Abeta increased spontaneous firing activity dose dependently, reaching the maximum after 1 hour of treatment, which was followed by a complete cessation of spikes following a few hours [42]. The discrepancy between this and our result may be due to the fact that the authors have used a cell culture largely devoid of glial elements, while we have used a complex microcircuitry built up from all of the neuronal cell types (including glia) forming elaborate connections.

The forms of NMDAR are heterotetramer including two NR1 and two NR2 subunits [43]. One particular subunit, NR2B, is mainly localized in extrasynaptic side. Abeta induced elevation of spiking activity could be prevented by blocking the NR2B subunits, suggesting that Abeta activates extrasynaptic NMDARs. The role of extrasynaptic or perisynaptic NMDARs in Abeta(1-42)-induced changes in functional synaptic plasticity and subsequent cell death has received much attention recently [22, 44, 45]. Extrasynaptic NMDARs contain predominantly NR2B subunits, which in contrast to NR2A-containing synaptic NMDARs trigger apoptotic signaling cascade [46]. Recently it was shown, using organotypic hippocampal cell cultures, that Abeta induces neuronal death and subsequent tau hyperphosporilation via extrasynaptic NR2B subunit [47]. Blocking NR2B subunits not only prevents hyperexcitation caused by Abeta but also rescues Abeta induced LTP impairment [22, 48], suggesting that blocking NR2B subunits might be a promising target in AD. However, others have reported that Abeta oligomers directly activate NMDARs especially via NR2A subunit [49, 50].

## 5. Conclusions

Taken together, we show that two electrophysiological events, recorded from hippocampal slices, which evoked fEPSPs and spontaneous firing, are not mediated by the same mechanisms. Evoked fEPSPs, but not firing activity, were mainly regulated by AMPARs. In contrast, spontaneous spikes are governed by NMDAR function. Bath applications of synaptotoxic Abeta(1-42) enhanced firing activity in an NR2B-dependent manner without altering evoked fEPSPs. These effects may contribute to synaptic dysfunctions seen in early AD.

## 6. Highlights

- Spontaneous discharges and evoked fEPSPs were recorded from the CA1 of murine slices.
- AMPA receptor blockade ablates fEPSPs without any effect on spiking rate.
- NMDA receptor modulation affects spontaneous spiking, but not evoked fEPSPs.
- Synaptotoxic amyloid-beta leaves evoked fEPSPs unaltered but increases firing activity via NR2B.
- These results confirm that amyloid-beta induces hyperexcitation through NR2B activation.

## Supplementary Material

Supplementary Figure 1. CNQX abolished evoked fEPSPs even under condition that promotes NMDAR activation (in low Mg^2+^ ACSF)
Supplementary Figure 2. Low concentration of AMPA (0.05 *μ*M) increased basal activity which hindered the effective detection of spikes. Figure shows representative traces before (left) and after (right) AMPA application.
Supplementary Figure 3. Small concentration of NMDA (0.5 *μ*M), which was reported to activate extrasynaptic NMDARs, did not affect fEPSPs
Supplementary Figure 4. Abeta(1-42) samples used for the recordings contained clearly detectable SDS-stable trimers (≈15 kDa) and higher molecular weight protofibrils. Staining with OC antibody, which is specific for species having beta-sheet conformation, shows that the small low-n oligomers are of prefibrillar nature.

## Abbreviations

Abeta(1-42): Amyloid-beta(1-42)
ACSF: Artificial cerebrospinal fluid
AD: Alzheimer's disease
AMPA: *α*-Amino-3-hydroxy-5-methyl-4-isoxazolepropionic acid
AMPAR: *α*-Amino-3-hydroxy-5-methyl-4-isoxazolepropionic acid receptor
ANOVA: Analysis of variance
CNQX: 6-Cyano-7-nitroquinoxaline-2,3-dione
fEPSP: Field excitatory postsynaptic potential
GABA: Gamma-aminobutyric acid
Glu: Glutamate
Low Mg^2+^ ACSF: Low magnesium concentration-containing artificial cerebrospinal fluid
LTP: Long-term potentiation
MEA: Multielectrode array
NMDA: N-Methyl-D-aspartate
NMDAR: N-Methyl-D-aspartate receptor
TTX: Tetrodotoxin
WB: Western blot.
