# Supplementary material for: Abeta(1-42) Enhances Neuronal Excitability in the CA1 via NR2B Subunit-Containing NMDA Receptors
Source: Neural Plast. 2014 Sep 3;2014:584314. doi: 10.1155/2014/584314 (PMC4168240; doi:10.1155/2014/584314)
Supplement: Supplementary file 1 — Supplementary Figure 1. CNQX abolished evoked fEPSPs even under condition that promotes NMDAR activation (in low Mg2+ ACSF) Supplementary Figure 2. Low concentration of AMPA (0.05 μM) increased basal activity which hindered the effective detection of spikes. Figure shows representative traces before (left) and after (right) AMPA application. Supplementary Figure 3. Small concentration of NMDA (0.5 μM), which was reported to activate extrasynaptic NMDARs, did not affect fEPSPs Supplementary Figure 4. Abeta(1-42) samples used for the recordings contained clearly detectable SDS-stable trimers (≈15 kDa) and higher molecular weight protofibrils. Staining with OC antibody, which is specific for species having beta-sheet conformation, shows that the small low-n oligomers are of prefibrillar nature. [file 584314.f1.pdf]

Supplementary material

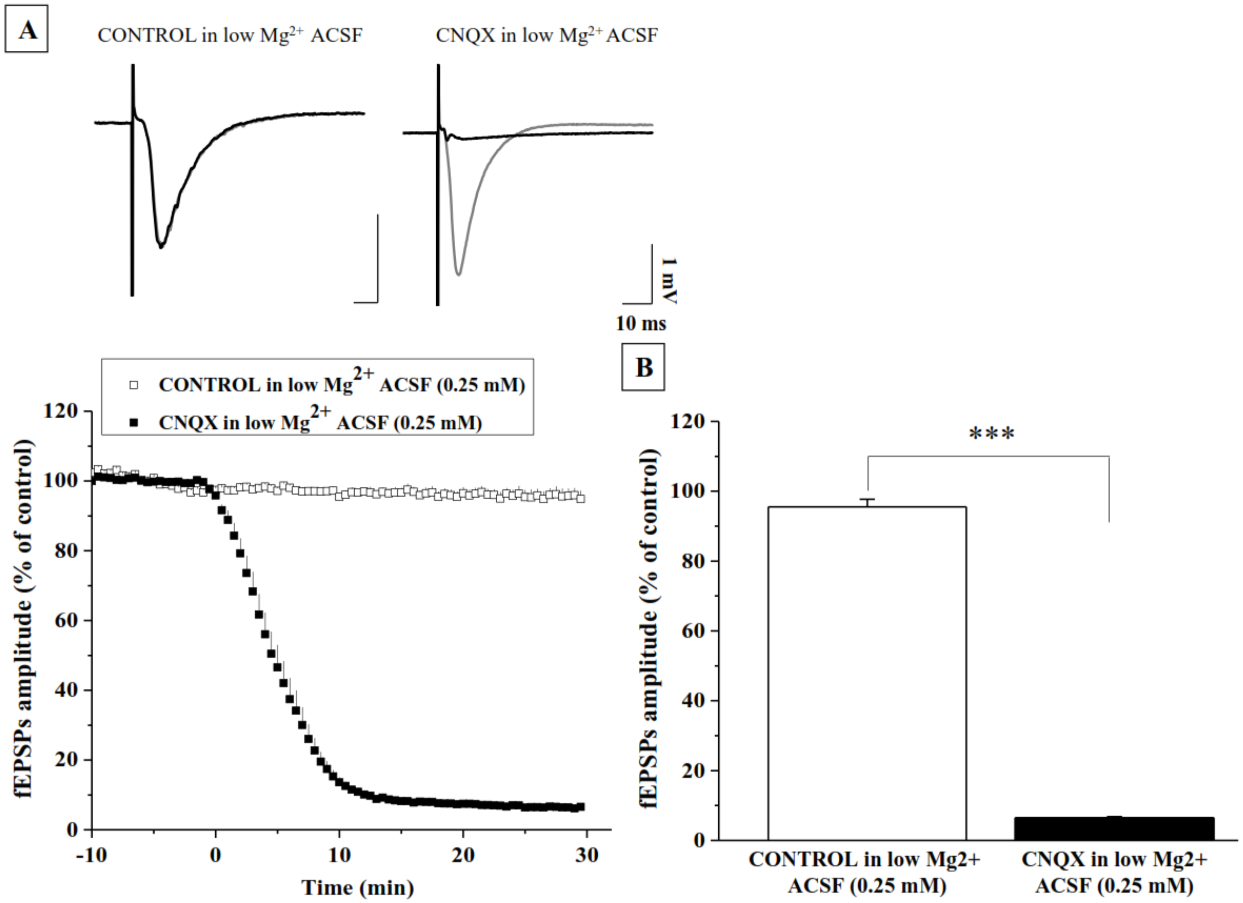

Supplementary Figure 1. CNQX abolished evoked fEPSPs even under condition that promotes NMDAR activation (in low Mg^{2+} ACSF) (**A**). Upper panel shows representative fEPSPs before (gray) and after (black) treatment. Bar graphs show the average of the fEPSPs amplitudes of the 25-30 min period after treatment (**B**). Error bars show SEM; *** $p \leq 0.001$.

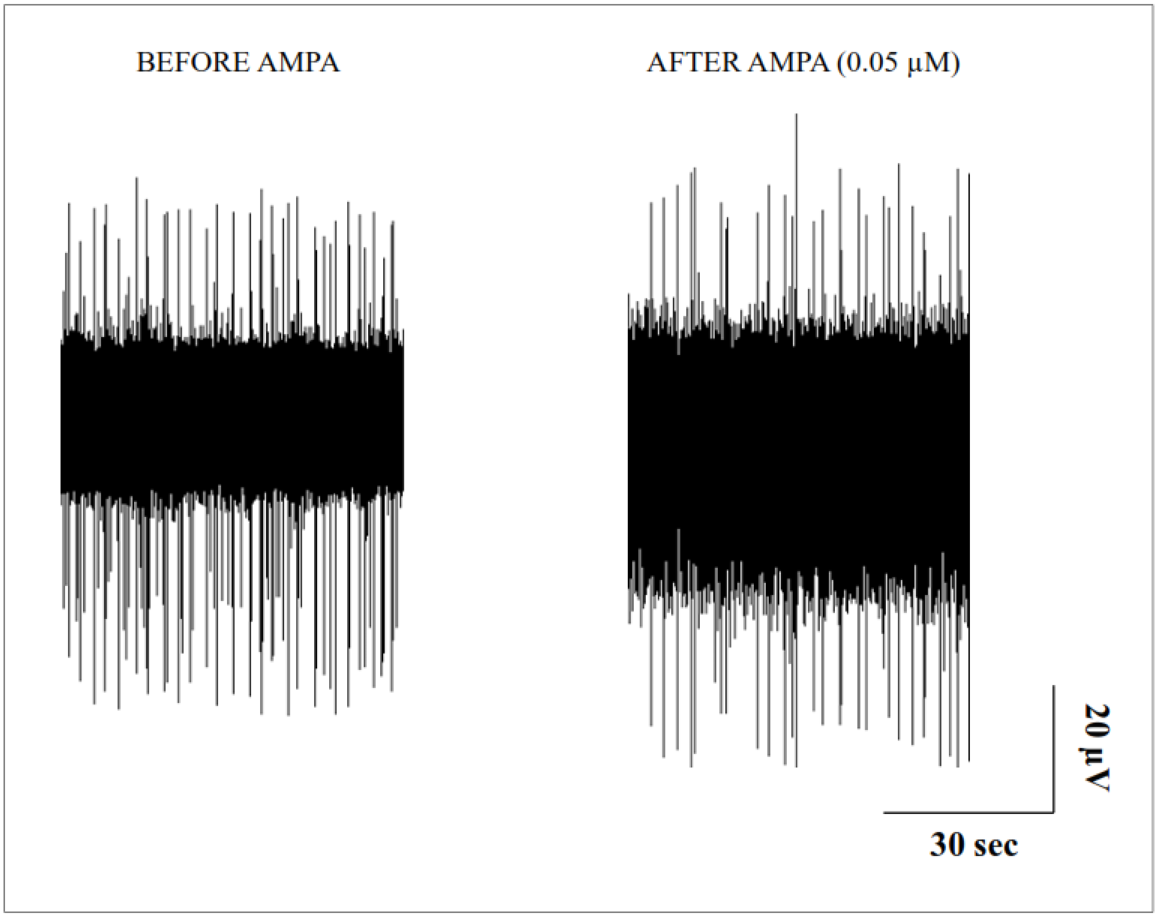

Supplementary Figure 2. Low concentration of AMPA (0.05 µM) increased basal activity which hindered the effective detection of spikes. Figure shows representative traces before (left) and after (right) AMPA application.

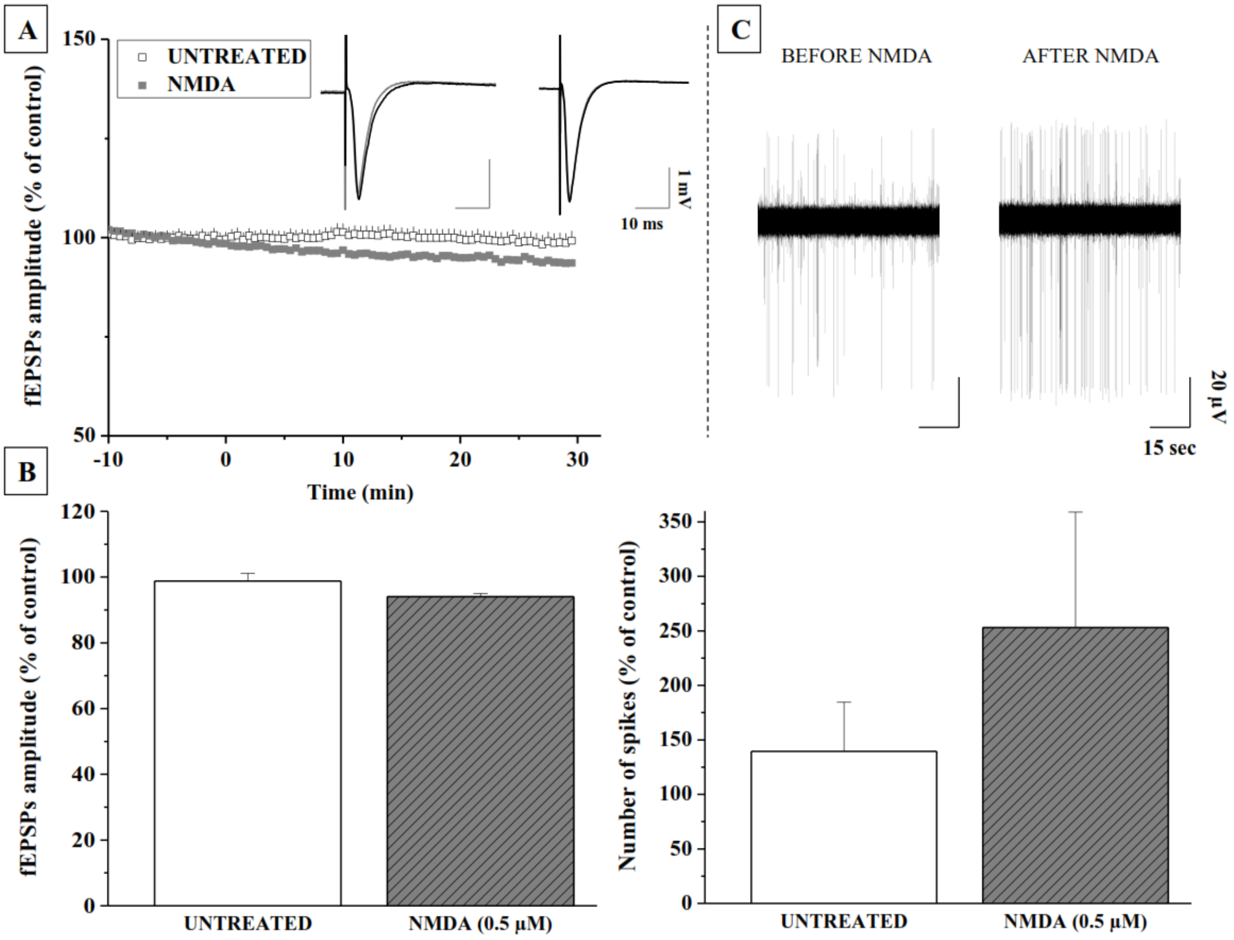

Supplementary Figure 3. Small concentration of NMDA (0.5 µM), which was reported to activate extrasynaptic NMDARs, did not affect fEPSPs (**A**). Inset shows representative fEPSPs before (grey) and after (black) treatment. Bar graphs show the average of the fEPSPs amplitudes of the 25-30 min period after treatment (**B**). Low concentration of NMDA elevated spiking frequency, although not significantly (**C**). Inset shows representative spike trains before anad after treatment. Error bars show SEM.

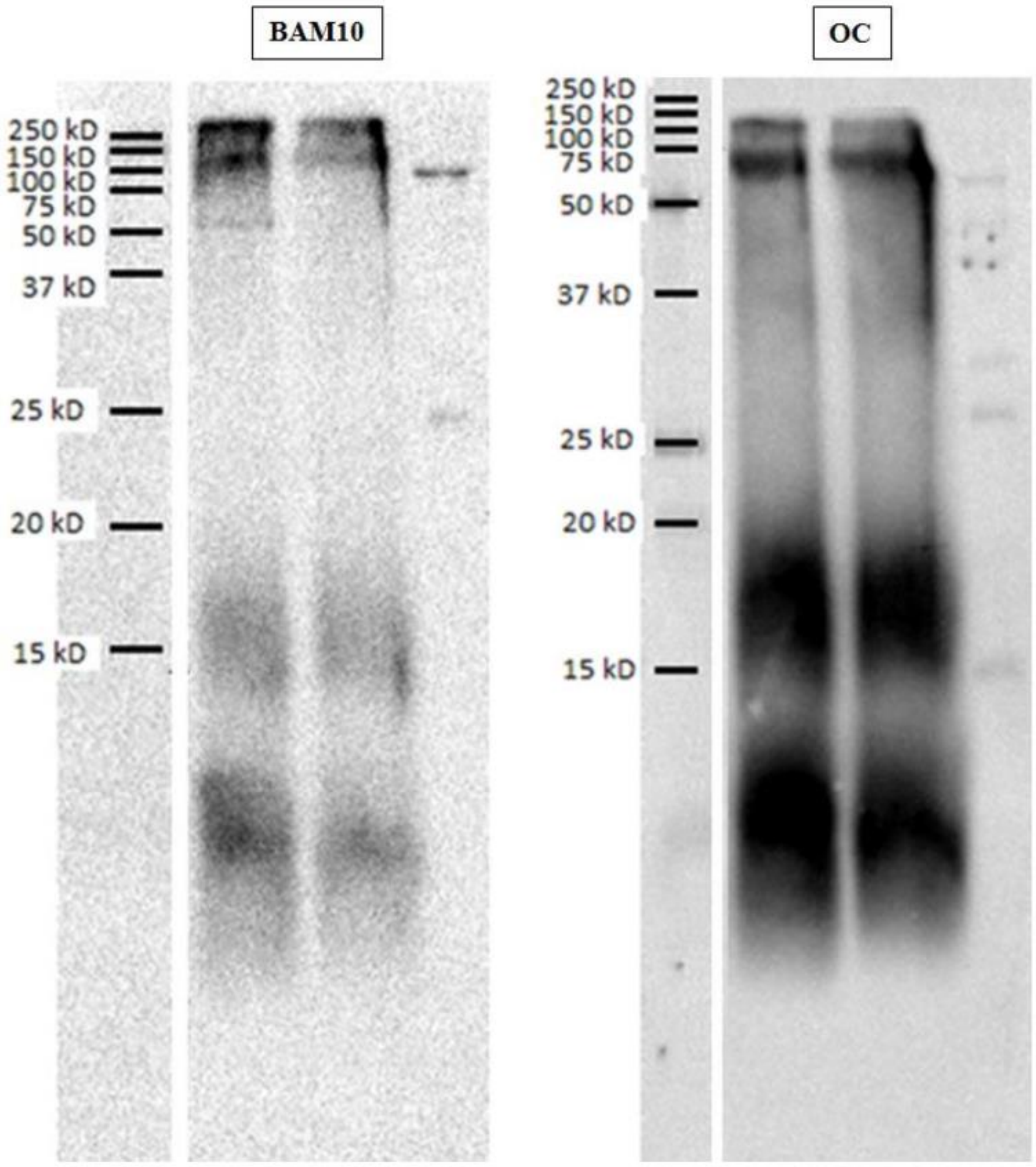

Supplementary Figure 4. Abeta(1-42) samples used for the recordings contained clearly detectable SDS-stable trimers (≈15 kDa) and higher molecular weight protofibrils. Staining with OC antibody, which is specific for species having beta-sheet conformation, shows that the small low-n oligomers are of prefibrillar nature.
